# Complete Genome and Plasmid Sequences of Three Canadian Isolates of *Salmonella enterica* subsp. *enterica* Serovar Heidelberg from Human and Food Sources

**DOI:** 10.1128/genomeA.01526-15

**Published:** 2016-01-14

**Authors:** Geneviève Labbé, Romaine Edirmanasinghe, Kim Ziebell, John H. E. Nash, Sadjia Bekal, E. Jane Parmley, Michael R. Mulvey, Roger P. Johnson

**Affiliations:** a National Microbiology Laboratory at Guelph, Public Health Agency of Canada, Guelph, Ontario, Canada; b Department of Medical Microbiology, University of Manitoba, Winnipeg, Manitoba, Canada; c National Microbiology Laboratory at the Canadian Science Centre for Human and Animal Health, Public Health Agency of Canada, Winnipeg, Manitoba, Canada; d National Microbiology Laboratory at Toronto, Public Health Agency of Canada, Toronto, Ontario, Canada; e Laboratoire de Santé Publique du Québec, Sainte-Anne-de-Bellevue, Québec, Canada; f Centre for Foodborne, Environmental and Zoonotic Infectious Diseases, Public Health Agency of Canada, Guelph, Ontario, Canada

## Abstract

Isolates of *Salmonella enterica* subsp. *enterica* serovar Heidelberg are often associated with poultry products and may cause severe human illness. Here, we report the fully assembled genome and plasmid sequences of three *S*. Heidelberg strains with phage types 9, 29, and 41.

## GENOME ANNOUNCEMENT

We present here the closed genome and plasmid sequences of three isolates of *Salmonella enterica* subsp. *enterica* serovar Heidelberg from Quebec, Canada, which include one human clinical isolate (12-4374, phage type 41 [PT41]) and two food isolates, one from turkey meat (N13-01290, PT9) and one from chicken meat (SA02DT10168701, PT29). The antimicrobial resistance profile of the turkey isolate includes amoxicillin-clavulanic acid, ampicillin, cefoxitin, ceftiofur, ceftriaxone, streptomycin, sulfamethoxazole, and tetracycline, and the profiles of the human and chicken isolates include amoxicillin-clavulanic acid, ampicillin, cefoxitin, ceftiofur, and ceftriaxone.

Genomic DNA was extracted using either the Qiagen EZ1 DNA tissue kit (Qiagen, Hilden, Germany), or the EpiCentre MasterPure complete DNA and RNA purification kit (Epicentre, Madison, WI)*.* Sequencing was performed on two platforms: (i) PacBio (at the Innovation Centre, at McGill University and Genome Quebec, Quebec, Canada, using 2 single-molecule real-time [SMRT] cells in an RSII sequencer), which generated 96,807 to 190,337 raw subreads averaging 4,769 to 5,306 bp in length with 92 to 167× coverage, and were assembled into contigs by the Innovation Centre using the HGAP workflow (1); and (ii) the Illumina MiSeq platform (at the Public Health Agency of Canada [PHAC] National Microbiology Laboratory, Winnipeg, Canada) with 2 × 251 paired-end runs after library preparation with the Illumina Nextera XT DNA library preparation kit, achieving 116 to 150× coverage. The Illumina reads were analyzed and quality checked using FastQC (http://www.bioinformatics.babraham.ac.uk/projects/fastqc/). Also, an optical map of the chicken isolate was generated using the restriction enzyme NcoI (OpGen, Inc., Gaithersburg, MD) and used to verify correct the contig assembly. Unweighted pair group method using average linkages (UPGMA) similarity clustering of the restriction fragments in the whole-genome optical map of the chicken isolate with *in silico* maps of publicly available *S*. Heidelberg isolates was performed using MapSolver version 2.1.1 (OpGen, Inc.). Genome assemblies were created by using the MIRA assembler version 4.9.3 (2) and by manually checking potential joins using the Gap5 software of the Staden package (3). A comparison of the genome assemblies with the genome optical map and with closely related plasmid sequences found in GenBank, together with the finishing process, produced fully assembled genomes and plasmids. To verify that no plasmids were missed, the nonmatched reads were used to produce *de novo* assemblies for each data set, and the remaining contigs were subjected to BLASTn searches and analyzed for gene content. The genomes consisted of single-chromosome contigs ranging from 4,751,447 to 4,809,628 bp, with an average G+C content of ~52.18%, and the plasmid contigs ranged from ~2,096 to 236,176 bp, with G+C content ranging from ~41.17 to 55.82%. The genomes and plasmids were annotated with the National Center for Biotechnology Information (NCBI) Prokaryotic Genome Annotation Pipeline (PGAP) (http://ncbi.nlm.nih.gov/genomes/static/Pipeline.html), identifying an average of ~4,550 coding DNA sequences (CDSs) per genome and ~2 to 270 CDSs per plasmid.

### Nucleotide sequence accession numbers.

The complete genome sequences of these three isolates of *S*. Heidelberg and their plasmids (Table 1) have been deposited in GenBank under BioProject number 298211. The GenBank accession numbers are listed in Table 1.

**TABLE 1:** Accession and isolate numbers for the genomes and plasmids of three *Salmonella* Heidelberg isolates sequenced in this study

GenBank accession no.	Local reference ID^a^	Original isolate no.	Phage type
CP012921	SA02DT10168701_Complete_Genome_4751447bp	SA02DT10168701	29
CP012922	pSA02DT10168701_37_Complete_Plasmid_37697bp		
CP012923	pSA02DT10168701_99_Complete_Plasmid_99011bp		
CP012924	12–4374_Complete_Genome_4790331bp	ID118758	41
CP012925	p12-4374_2_Complete_Plasmid_2096bp		
CP012926	p12-4374_37_Complete_Plasmid_37697bp		
CP012927	p12-4374_3_Complete_Plasmid_3372bp		
CP012928	p12-4374_62_Complete_Plasmid_62920bp		
CP012929	p12-4374_96_Complete_Plasmid_96042bp		
CP012930	N13-01290_Complete_Genome_4809628bp	SA02TK12002101	9
CP012931	pN13-01290_23_Complete_Plasmid_236176bp		
CP012932	pN13-01290_2_Complete_Plasmid_2096bp		
CP012933	pN13-01290_3-1_Complete_Plasmid_3319bp		
CP012934	pN13-01290_3-2_Complete_Plasmid_3372bp		
CP012935	pN13-01290_3-3_Complete_Plasmid_3905bp		
CP012936	pN13-01290_98_Complete_Plasmid_98999bp		

a ID, identification.
